# Regulation of Carbohydrate Metabolism by Trehalose-6-Phosphate Synthase 3 in the Brown Planthopper, *Nilaparvata lugens*

**DOI:** 10.3389/fphys.2020.575485

**Published:** 2020-09-17

**Authors:** Sha-Sha Wang, Guo-Yong Li, Yong-Kang Liu, Yu-Jia Luo, Cai-Di Xu, Can Li, Bin Tang

**Affiliations:** ^1^Guizhou Provincial Key Laboratory for Rare Animal and Economic Insect of the Mountainous Region, Department of Biology and Engineering of Environment, Guiyang University, Guiyang, China; ^2^College of Life and Environmental Sciences, Hangzhou Normal University, Hangzhou, China

**Keywords:** delphacidae, trehalose-6-phosphate synthase, trehalose metabolism, glycogen, RNA interference

## Abstract

*Nilaparvata lugens* (Stål) (Hemiptera: Delphacidae) is one of the pests that harm rice. In this paper, a new trehalose-6-phosphate synthase gene, *TPS3*, was identified by transcriptome sequencing and gene cloning. To explore its role in the energy metabolism of *N. lugens* we examined the carbohydrate contents at different stages of development, the tissue expression of *TPS*, and some physiological and biochemical indicators by injecting dsTPS3 and dsTPSs (a proportional mixture of dsTPS1, dsTPS2, and dsTPS3). The glucose content at the fifth instar was significantly higher than that in the fourth instar and the adult stages. The trehalose and glycogen contents before molting were higher than those after molting. *TPS1*, *TPS2*, and *TPS3* were expressed in the head, leg, wing bud, and cuticle, with the highest expression in the wing bud. In addition, compared with the control group, the glucose content increased significantly at 48 h after RNA interference, and the trehalose content decreased significantly after 72 h. qRT-PCR showed that the expression level of *UGPase* decreased significantly at 48 h after injection, whereas *GS* expression increased significantly at 48 h after injecting dsTPS3. After dsTPS injection, the expression levels of *PPGM2*, *UGPase*, *GP*, and *GS* increased significantly at 72 h. After interfering with the expression of *TPS3* gene alone, *UGPase* expression decreased significantly at 48 h, and *GS* expression increased significantly at 72 h. Finally, combined with the digital gene expression and pathway analysis, 1439 and 1346 genes were upregulated, and 2127 and 1927 genes were downregulated in the dsTPS3 and dsTPSs groups, respectively. The function of most differential genes was concentrated in sugar metabolism, lipid metabolism, and amino acid metabolism. The results indicated that *TPS3* plays a key role in the energy metabolism of *N. lugens* and confirmed that *TPS3* is a feasible target gene for RNA interference in *N. lugens*. Simultaneously, they provide a theoretical basis for the development and utilization of *TPS3* to control pests.

## Introduction

Crops are often attacked by various agricultural pests, resulting in decreased yield and quality of agricultural products. Therefore, agricultural pests are one of the factors that constrain global agricultural development. Nearly half the global population consumes rice, especially Chinese residents (Sun et al., 2019). Locusts can pose a major threat to rice, and destruction of produce by locusts occurs in frequent bursts. Among them, *Nilaparvata lugens* (Stål) (Hemiptera: Delphacidae) is one of the most destructive pests of rice (Yang et al., 2019). This insect can use its probe to pierce the sheath phloem sap and absorb vascular fluids containing sucrose, amino acids, potassium, and ATP from vascular bundles (Hayashi and Chino, 1990). In addition to extracting nutrients from the vascular bundle, it can transmit viruses such as rice ragged stunt virus (RRSV) and rice grassy stunt virus to rice plants (Hibino, 1996; Wang et al., 2016). The use of large amounts of chemical pesticides is well known to the human body and the environment. It would be great to develop a new type of green pesticide to control pests by blocking the energy metabolism of planthoppers.

For all organisms, continuation of life is inseparable from energy metabolism. In mammals, glucose is the energy source and metabolic intermediate of living cells and is called the “fuel of life.” Glucose is also an important source of energy in insects. After feeding, the carbohydrates in food are absorbed by cells and produce energy through the glycolytic pathway; the excess glucose in circulation is stored in the form of trehalose and glycogen (Roach et al., 2012; Yamada et al., 2018). Glycogen is synthesized and stored in several tissues, including the muscles and fat body (Yamada et al., 2018). Glycogen metabolism is controlled by two enzymes, glycogen synthase (GS) and glycogen phosphorylase (GP). GP catalyzes the rate-limiting cleavage of glucose monomers at the ends of glycogen branches (Roach et al., 2012; Zhang et al., 2017; Yamada et al., 2019). In addition to glycogen, the concentration of the non-reducing disaccharide, trehalose, in insect hemolymph is maintained at a high level (Becker et al., 1996; Shukla et al., 2015). At the beginning of the 19th century, Wiggers first discovered this sugar in rye ergot; it was subsequently reported in mushrooms (Elbein et al., 2003; Shukla et al., 2015). Trehalose was also found in bacteria, plants, insects, and other organisms (Argüelles, 2014; Lunn et al., 2014; Shukla et al., 2015). Trehalose plays a variety of important roles in these organisms; for example, it acts as a structural component in bacterial cell walls, as a growth regulator and carbon source in plants and fungi, as an energy source in insects, and as a compatible solute against environmental stress (Crowe et al., 1984; Asano, 2003). In insects, trehalose is the main hemolymph sugar and is synthesized in the fat body. Two enzymes are involved in this synthetic pathway, namely trehalose-6-phosphate synthase (TPS) and trehalose 6-phosphate phosphatase (TPP). TPS catalyzes glucose transfer from uridine diphosphate glucose (UDP-glucose) to glucose-6-phosphate to form trehalose-6-phosphate (T-6-P) and UDP, whereas TPP dephosphorylates T-6-P to trehalose and inorganic phosphate (Shukla et al., 2015).

Trehalose-6-phosphate synthase genes have been cloned in many insects such as *Apis mellifera*, *Tribolium castaneum*, *Musca domestica*, and *Heortia vitessoides* (Chen et al., 2018, 2020; Łopieńska-Biernat et al., 2018; Zhang D. W. et al., 2019). As a tool for knocking out individual gene expression after transcription, RNA interference (RNAi) has been used to control the expression of specific genes in many experimental organisms (Mello and Conte, 2004; Han, 2018). A large number of studies on *TPS* have shown that *TPS* plays an important role in the growth and development of organisms. For example, when *TPS* expression in insects was inhibited, insects showed death and deformity phenotypes, indicating that *TPS* affects insect energy metabolism and chitin metabolism (Xiong et al., 2016; Chen et al., 2018, 2020). In addition, *TPS* acts as an inducible anti-stress gene that takes part in immune defense in *M. domestica* via synthesizing its product trehalose (Zhang D. W. et al., 2019). *TPS1* and *TPS2* have been reported in *N. lugens*, and related studies have been carried out on these genes (Yang et al., 2017). In this study, we cloned a new *TPS* named *TPS3*. This study used RNAi to explore the effects of *TPS3* gene on energy metabolism, and to evaluate whether *TPS3* has the theoretical potential as a target gene for controlling brown planthoppers.

## Materials and Methods

### Insect and Material Collection

The brown planthoppers (*N. lugens*) collected from the Zhejiang Academy of Agricultural Sciences were kept in the laboratory of the author. The brown planthopper and rice were tested and cultivated in an artificial climate chamber. Brown planthopper feeding conditions: temperature 26 ± 1°C, photoperiod 16 h: 8 h (light: dark), relative humidity 70%.

The rice variety used to raise the brown planthopper was TN1 (Taichung Native 1). Rice planting steps: First, the rice seeds were soaked in warm water at about 70°C for 10 min, followed by soaking in tap water and placing them in an artificial climate incubator at 30°C for 24 h. Then, the water was drained, rinsed with tap water, and the seeds were wrapped with wet gauze, and placed in a 30°C artificial climate incubator for 24–48 h. After the seeds germinated, we planted them in plastic pots and fertilized them appropriately. After the seedlings grew to about 10 cm, they were transferred to the field for cultivation. After rice was grown to the middle of the tillering, it was moved to the insect cage; the rice plants were replaced every 2–3 days.

From the fourth instar nymph to the third day of the adult, the developmental expression materials of the brown planthopper were collected every 12 h. Three biological replicates were performed at each developmental stage, and seven planthoppers were collected for each replicate. The expression material of the brown planthopper tissue was taken from the adult stage and dissected under the Leica EZ4 dissecting microscope to obtain the planthopper head, foot, wing bud, and epidermis. Three biological replicates were performed for each tissue material, and more than 200 individuals were collected for each replicate.

### RNA Extraction and cDNA Synthesis

Total RNA was isolated from whole brown planthopper using TRIzol reagent (Invitrogen, Carlsbad, CA, United States) according to the method described by Yang et al. (2017). After extraction, the RNA quality was first determined by agarose gel electrophoresis, and the purity and concentration of the isolated RNA were determined spectrophotometrically using a NanoDrop^TM^ 2000 (Thermo Scientific, Wilmington, United States). The remaining RNA was stored in a −80°C freezer. The first strand of cDNA was synthesized using the TaKaRa PrimeScript^TM^ RT reagent Kit with the gDNA Eraser kit (TaKaRa, Dalian, China) that includes the removal reaction and reverse transcription reaction of genomic DNA. The specific experimental method can be found in the kit manual. The cDNA was stored in a refrigerator at −20°C.

### Double-Stranded RNA Synthesis

First, the dsRNA primer of *N. lugens TPS3* (GenBank: KU55 6827.1) gene was designed using Primer Premier 5 software. The T7 sequence was 5′-GGATCCTAATACGACTCACTATAGG-3′. In addition, the dsRNA primer of *GFP*, *TPS1* (GenBank: GQ397450.1), and *TPS2* (GenBank: KU556826.1) genes were used as described by Yang et al. (2017). Specific primer sequences are shown in Table 1.

**TABLE 1:** Primers used for dsTPS1, dsTPS2, dsTPS3 and dsGFP.

Primer name	Primer Sequence (5′-3′)
dsNlTPS1-F	ACCAGGAGTTGAAGGAGGAG
dsNlTPS1-R	GCTATGGGCACCCTGATC
dsNlTPS1-FT	GGATCCTAATACGACTCACTATAGGACCAGGAGTTGAAGG AGGAG
dsNlTPS1-RT	GGATCCTAATACGACTCACTATAGGGCTATGGGCACCCT GATC
dsNlTPS2-F	CACCAAAGGTCTAAGGCACA
dsNlTPS2-R	TCCCTACGAGATCAACGATG
dsNlTPS2-FT	GGATCCTAATACGACTCACTATAGGCACCAAAGGTCTAAG GCACA
dsNlTPS2-RT	GGATCCTAATACGACTCACTATAGGTCCCTACGAGATCA ACGATG
dsNlTPS3-F	GAGTCTGACCTGATAGCCTTTA
dsNlTPS3-R	ATCGGAGTCCATTTAGTTGT
dsNlTPS3-FT	GGATCCTAATACGACTCACTATAGGGAGTCTGACCTGATAGC CTTTA
dsNlTPS3-RT	GGATCCTAATACGACTCACTATAGGATCGGAGTCCATTTA GTTGT
dsNlGFP-F	AAGGGCGAGGAGCTGTTCACCG
dsNlGFP-R	CAGCAGGACCATGTGATCGCGC
dsNlGFP-FT	GGATCCTAATACGACTCACTATAGGAAGGGCGAGGAGCTGT TCACCG
dsNlGFP-RT	GGATCCTAATACGACTCACTATAGGCAGCAGGACCATGTGA TCGCGC

The T7 RiboMAX^TM^ Express RNAi System is an *in vitro* transcription system that synthesizes milligrams of dsRNA in a short period of time. Moreover, the synthesized dsRNA does not contain the contamination of proteins and other components, and it was suitable for RNAi of the brown planthopper *TPS* gene in this study. The two complementary RNA strands were synthesized using plasmid DNA as a template, and the single-stranded RNA then generates double-stranded RNA after annealing. The DNA template and single-stranded RNA remaining in the reaction are removed by nuclease digestion, and the resulting dsRNA is purified by precipitation with isopropanol to obtain a dsRNA that can be used for RNAi experiments.

### Microinjection

The injection volume was 200 ng of dsRNA per brown planthopper, and the injection was performed in the fifth instar nymph of brown planthopper, and the injection site was the lateral epidermis between the two pairs of hind limbs in the thorax of the brown planthopper. The number of injections per treatment group was 50–100 planthoppers, and three biological replicates were performed. The microinjection system was first adjusted, the nitrogen pressure was adjusted to a suitable scale, and the volume of each dsRNA injected was determined under a microscope using standard capillaries. The brown planthoppers were then immobilized with carbon dioxide, and placed ventral-side up on a pre-prepared agarose plate. The insect was then injected under a microscope, and successfully injected brown planthoppers were transferred to a glass tube containing fresh rice for subsequent experiments.

### Quantitative Real-Time PCR

The primers required for Quantitative Real-time PCR (qRT-PCR) were first designed using Primer Premier 5 software. In this experiment, the brown planthopper *18S* gene was used as an internal reference gene (Yang et al., 2017). The detailed qRT-PCR primers are shown in Table 2.

**TABLE 2:** Primers used for qRT-PCR.

Primer name	Forward Primer (5′-3′)	Reverse Primer (5′-3′)
QNl18S QNlTPS1 QNlTPS2 QNlTPS3	CGCTACTACCGATTGAA AAGACTGAGGCGAATGGT AGAGTGGACCGCAACAACA GTGATGCGTCGGTGGCTAT	GGAAACCTTGTTACGACTT AAGGTGGAAATGGAATGTG TCAACGCCGAGAATGACTT CCGTTCATCATTGGGCATAGT
QNlG-6-pase	TTTCGGCTCACTTCCCTC	GCAGTAATCAACATAGCACCT
QNlPPGM1	TAAAGCCAGTCATACCAG	TCGATAGATAGATTGAGGC
QNlPPGM2	ACAGCCAGTCACAATCCG	GGTAACAGCATCTGGAGC
QNlUGPase	GCACGGTGACTTCTACGA	TGAGGTCAACTGTGGCTC
QNlGP	GCTGCCTATGGCTATGGTATTC	TCTGAGTGTTGACCCACTTCTTG
QNlGS	GCTCCAAAGCCTATGTTTCTACT	TGGTAACCCCTGTCCCTCA

qRT-PCR system preparation: SYBR Premix Ex Taq (10 μL), template cDNA (1 μL), forward primer (1 μL), reverse primer (1 μL); the volume was made up to 20 μL with sterile water. qRT-PCR system: pre-denaturation at 95°C for 3 min, denaturation at 95°C for 5 s and annealing at 55–60°C for 30 s (39 cycles), and a dissolution curve at 95°C for 5 s. The CT values of several genes were determined by qRT-PCR, then, the relative expression of target genes was calculated by the 2^–ΔΔ*CT*^ method (Livaka and Schmittgenb, 2001). All comparisons were performed with three biological replicates and three technical replicates.

### Determination of Carbohydrate Contents

#### Material Handling Process

Seven individuals were placed in a mortar and ground with liquid nitrogen. The ground material was transferred to a 1.5 mL Eppendorf tube and 200 μL of phosphate-buffered saline (PBS, 20 mM, pH 6.0) was added and then sonicated in a sonicator. After complete crushing, 800 μL of PBS was added to the Eppendorf tube, and the material was centrifuged (4°C, 1000 × *g*) for 20 min. A portion of the supernatant after centrifugation was added to a new Eppendorf tube, and subjected to ultracentrifugation (4°C, 20800 × *g*) for 1 h; another portion was used to detect the trehalose content, glycogen content and protein content. The supernatant after the second centrifugation was used to determine the glucose content and protein content.

The anthrone method was used to measure the trehalose content in brown planthoppers. The specific experimental procedure is as follows: 10 μL of the test solution or trehalose standard solution was placed in a 1.5 mL Eppendorf tube. The trehalose concentrations used to generate the standard curve were 1.6, 0.8, 0.6, 0.4, 0.2, 0.1, and 0.05 mM. Next, 10 μL of 1% H_2_SO_4_ was added to each sample, and then placed in a water bath at 90°C for 10 min. After incubation in the water bath, the samples were placed on ice for 3 min, and 10 μL of 30% KOH was added. The mixture was then placed in a water bath at 90°C for 10 min, and cooled on ice for 3 min. Next, 200 μL of the developer (0.02 g anthrone dissolved in 80% H_2_SO_4_) was added to the EP tube and placed in a water bath at 90°C for 10 min. Finally, the samples were placed on ice for cooling, and absorbance at 630 nm was measured using a microplate reader. The glycogen content and glucose content were measured using the Glucose (GO) Assay Kit from Sigma-Aldrich. The test solution was first reacted with 0.1 U/L of starch transglucosidase at 40°C for 4 h, so that the glycogen was first converted to glucose, and the glucose content was then measured in the same manner. The assay was carried out according to the specified method (Yang et al., 2017). The protein concentration was determined using BCA protein assay kit (Pierce Biotechnology, Rockford, IL, United States). According to the number of samples, add 50 volumes of BCA A reagent to 1 volume of BCA B reagent to prepare an appropriate amount of BCA. Then, when the protein standard solution was completely dissolved, take out 10 μL of the standard solution and dilute it to 100 μL with PBS to make the final concentration 0.5 mg/mL. According to the kit instructions, prepare standard solutions of different concentrations to obtain a standard curve. Then add 200 μL of BCA to 20 μL of each sample solution and standard solution. After placing them in a constant temperature incubator at 37°C for 30 min, the absorbance was measured at 562 nm. The above experiments were carried out three biological replicates and three technical replicates.

### DGE Sequencing and Analysis

The extracted RNA samples were sent to the Beijing Genomics Institution (BGI) for DGE sequencing. The instrument used was Hiseq2000 (Illumina, United States).

Brown planthoppers injected with dsGFP were used as the control group, and those in the experimental groups were injected with dsTPS3 and dsTPSs. Combined with the sequencing data from the previous study, the differential genes were screened by comparative analysis of sequencing data from the control group and the experimental group. The *P* value was then corrected by a multiple hypothesis test, and the threshold of the *P* value was controlled by the False Discovery Rate (FDR). In this study, only genes with an FDR less than or equal to 0.001 and in multiples of difference greater than or equal to 2 were selected for screening the differential genes.

### Data Analysis

The data presented in the final result figures are expressed as the mean + standard error (SE) of three biological replicates. Differences were analyzed using Tukey’s test of One-way ANOVA in IBM SPSS Statistics 20 (^∗∗^*P* < 0.01; ^∗^*P* < 0.05). The analyzed data were plotted using the Grouped Error Bars chart option in the Vertical Bar Chart type of SigmaPlot 10.0 software.

## Results

### Developmental Expression Pattern of Carbohydrates

**FIGURE 1:**
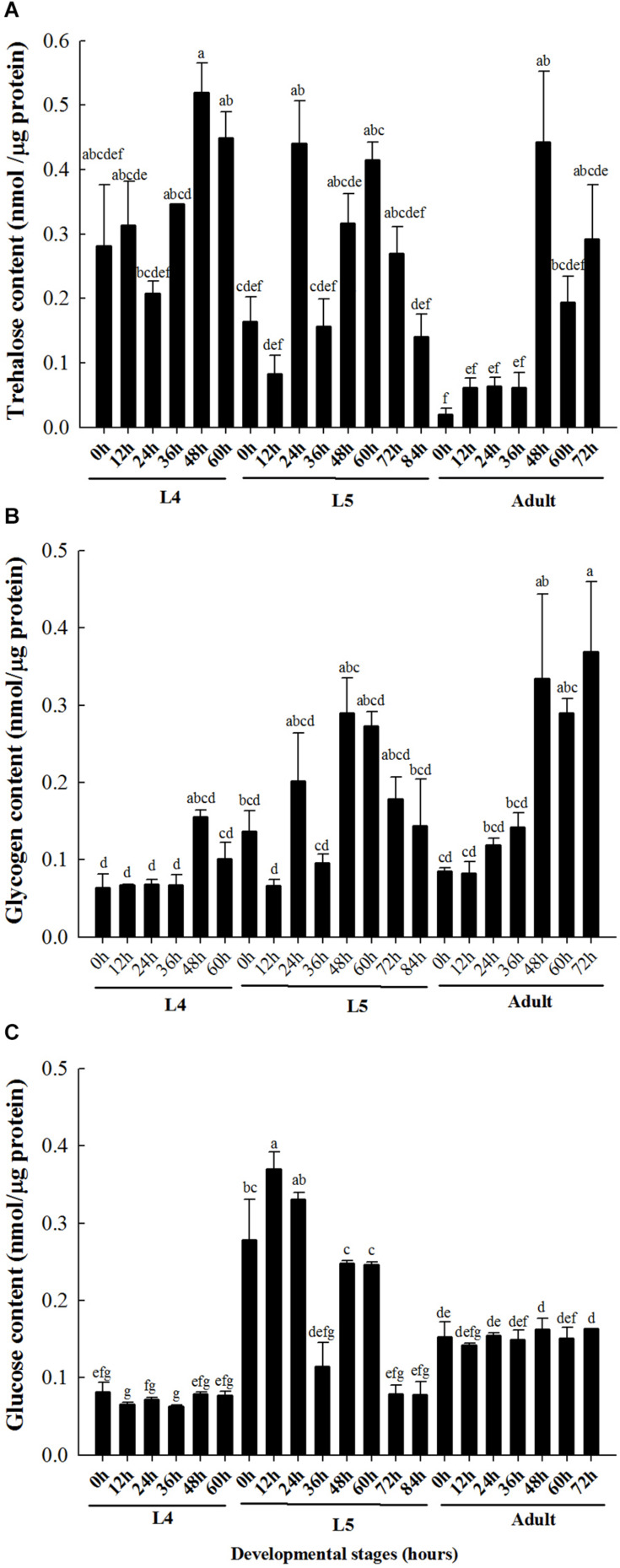
Carbohydrate contents in different developmental stages of brown planthopper. **(A)** Trehalose content. **(B)** Glycogen content. **(C)** Glucose content. L4 indicates fourth instar larva and L5 indicates fifth instar larva. Each bar depicts the mean (+SE) of three biological replicates, with different letters above the error bars denoting significant differences between the means (Tukey’s test, α = 0.05; each group was analyzed separately).

The content of trehalose in the brown planthopper at 24 h in the fourth instar was lower than that at 48 h in the fourth instar (Figure 1A). At the fifth instar stage, the trehalose content at 24 h was significantly higher than that at 0, 12, 36, and 84 h (Figure 1A). For adults after emergence, the trehalose and glycogen contents were relatively low in the initial stage (0 to 36 h), but both sugar contents increased significantly after 48 h (Figures 1A,B). Finally, the glucose content was significantly higher than that at the fourth instar and adult stage in the 0–60 h phase of the fifth instar, and the glucose content in the adult stage was higher than that in the fourth instar stage (Figure 1C).

### Tissue Expression Pattern of *TPS* Genes in *N. lugens* Adults

**FIGURE 2:**
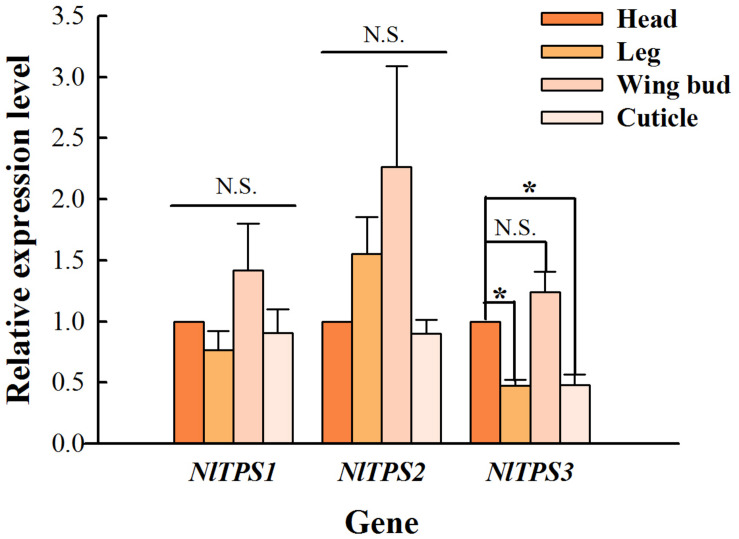
Comparison of changes in relative expression levels of the three *TPS* genes in the adult tissues of brown planthopper. The expression levels of these genes were determined by qRT-PCR using the housekeeping gene *18S* as a reference. Each bar depicts the mean (+SE) of three biological replicates. The relative expression level of each gene in the head was used as the control (Tukey’s test; *P < 0.05, N.S. means no significant difference).

The expression level of *TPS* in the head was used as a control. As seen in Figure 2, the expression levels of three *TPS* genes in the wing bud group were higher than those in other tissues, and the expression level of *TPS3* in the leg and cuticle was significantly lower than that in the control group.

### The Inhibitory Effect of dsRNA

**FIGURE 3:**
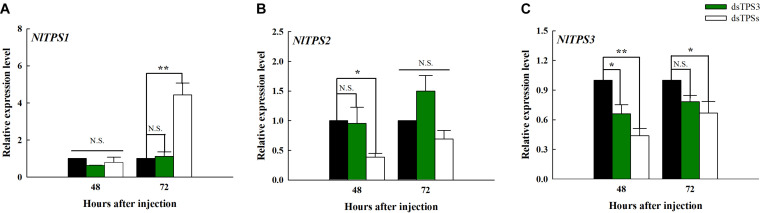
Changes in the expression levels of the three *TPS* genes after RNAi at 48 and 72 h in the fifth instar nymph of brown planthopper. The relative expression levels of trehalose-6-phosphate synthase 1 gene (*TPS1*) **(A)**, trehalose-6-phosphate synthase 2 gene (*TPS2*) **(B)**, and trehalose-6-phosphate synthase 3 gene (*TPS3*) **(C)**. Each bar depicts the mean (+SE) from three biological replicates. The relative expression level of each gene after injection of dsGFP was used as the control (Tukey’s test; **P < 0.01, *P < 0.05, N.S. means no significant difference).

The expression level of *TPS1* was significantly different from that of the control group only 72 h after the injection of dsTPSs, which showed an increase (Figure 3A). It can be seen from Figure 3B that when dsTPSs was injected for 48 h, the expression level of *TPS2* decreased significantly. When dsTPS3 or dsTPSs was injected into *N. lugens* for 48 h, the expression level of *TPS3* reduced by qRT-PCR detection (Figure 3C). Detecting the expression level at 72 h found that the expression level of *TPS3* in the dsTPS3 group was not significantly different from the control group, while the expression level of *TPS3* in the dsTPSs group was lower than that in the control group (Figure 3C).

### Comparison of Differential Genes After RNAi

**FIGURE 4:**
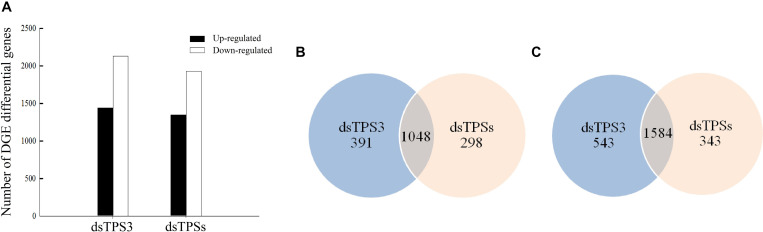
The number of differentially expressed genes after dsTPS3 and dsTPSs injection compared to dsGFP. **(A)** The differential expression of single genes in the two DGE databases is plotted according to each treatment. **(B,C)** Comparison of the number of upregulated **(B)** and downregulated **(C)** unigenes from the two DGE databases for each of the treatments. Blue: the first DGE-seq database only; apricot: the second DGE-seq database only; overlap: both databases.

The results of DGE analysis showed that after dsTPS3 injection, 1439 genes were upregulated, and 2127 genes were downregulated. When dsTPSs were injected, 1346 genes were upregulated, and 1927 genes were downregulated (Figure 4A). Comparing the two injection groups, 1048 and 1584 genes were identical in the upregulated and downregulated genes, respectively (Figure 4B).

### Significant Pathway Enrichment Analysis After RNAi

**FIGURE 5:**
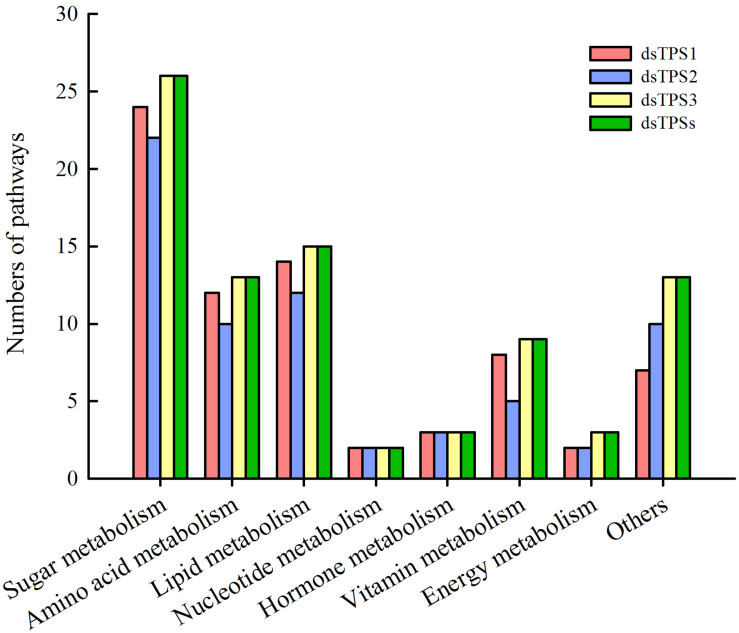
Pathway enrichment analysis after RNAi. Red: dsTPS1 injection group; Blue: dsTPS2 injection group; Yellow: dsTPS3 injection group; Green: dsTPSs injection group.

The results showed that more than 20 genes were involved in sugar metabolism, followed by more genes involved in amino acid metabolism and lipid metabolism. In addition, the genes involved in nucleotide metabolism, energy metabolism, and hormone metabolism are fewer than other pathways. Moreover, there are more than five genes in each group, and the functions of 15 or less genes are unknown (Figure 5).

### Changes in Carbohydrate Content After RNAi

**FIGURE 6:**
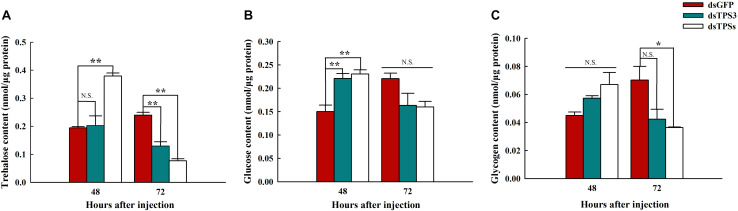
Trehalose, glycogen, and glucose contents after RNAi at 48 and 72 h in the fifth instar nymph of brown planthopper. **(A)** Trehalose content. **(B)** Glucose content. **(C)** Glycogen content. All *Nilaparvata lugens* larvae were divided into three groups and injected with dsGFP, dsTPS3, or dsTPSs. Each bar depicts the mean (+SE) from three biological replicates. The carbohydrate content at 48 or 72 h after dsGFP injection was used as the control (Tukey’s test; **P < 0.01, *P < 0.05, N.S. means no significant difference).

After knocking down the single *TPS3* gene, the trehalose content was decreased significantly at 72 h (Figure 6A), and the glucose content in *N. lugens* was increased significantly at 48 h (Figure 6B). In addition, the trehalose and glucose contents were increased significantly at 48 h (Figures 6A,B), and the trehalose and glycogen contents were decreased significantly at 72 h after mixed interference with the *TPS1*, *TPS2*, and *TPS3* genes (Figures 6A,C).

### Changes in the Expression Levels of Related Genes After RNAi

**FIGURE 7:**
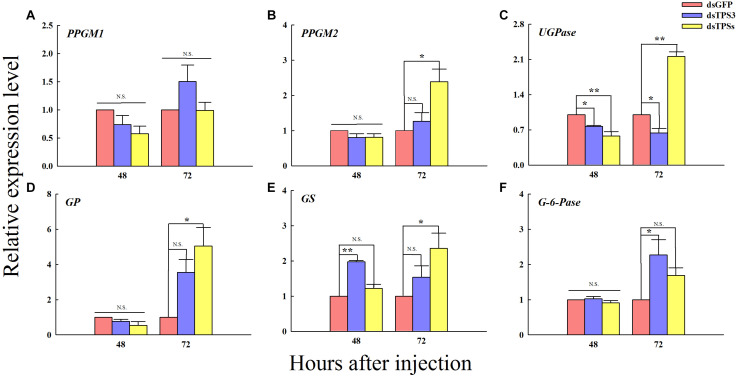
Changes in the expression levels of related genes after RNAi at 48 and 72 h in the fifth instar nymph of brown planthopper. The relative expression levels of phosphoglucomutase 1 gene (*PPGM1*) **(A)**, phosphoglucomutase 2 gene (*PPGM2*) **(B)**, UDP-Glucose pyrophosphorylase gene (*UGPase*) **(C)**, glycogen phosphorylase gene (*GP*) **(D)**, glycogen synthase gene (*GS*) **(E)**, and glucose-6-phosphatase gene (*G-6-Pase*) **(F)**. Each bar depicts the mean (+SE) from three biological replicates. The relative expression level of each gene after injection of dsGFP was used as the control (Tukey’s test; **P < 0.01, *P < 0.05, N.S. means no significant difference).

The relative expression levels of UDP-glucose pyrophosphorylase (UGPase) was decreased after 48 h of dsTPS3 and dsTPSs injection (Figure 7C). However, the relative expression level of *GS* was significantly increased after 48 h of interference with the expression of the *TPS3* gene alone (Figure 7E). The relative expression levels of phosphoglucomutase 2 gene (*PPGM2*), *UGPase*, GP, and GS were significantly increased at 72 h after mixed interference with *TPS1*, *TPS2*, and *TPS3* genes (Figures 7B–E). At 72 h after dsTPS injection, the expression level of UGPase was significantly decreased (Figure 7C), while the glucose-6-phosphatase gene (*G-6-Pase*) was increased significantly (Figure 7F).

## Discussion

Similar to glycogen metabolism, trehalose metabolism is controlled by two enzymes: TPS and trehalase (TRE) (Elbein et al., 2003). The carbohydrate metabolic rate is determined by the energy demand and is regulated by hormones (Arrese and Soulages, 2010). In mammals, the main nutrient in blood is glucose, and studies have determined that it is regulated by several hormones such as insulin and glucagon (Mochanová et al., 2018). The nutrient levels in the hemolymph of insects are mainly controlled by adipokinetic hormone (AKH) (Mochanová et al., 2018). Other hormones including octopamine, ecdysone, and insulin-like growth factor can regulate carbohydrate metabolism (Lorenz and Gäde, 2009; Arrese and Soulages, 2010; Kim and Neufeld, 2015; Jiang et al., 2017; Song et al., 2017; Kawabe et al., 2019). *A. mellifera* is a unique model system for aging and longevity studies. As a complete metamorphosis insect, the entire developmental period includes the egg, larva (age 1–7), pupa, and adult stages (Lu et al., 2018). Studies on *A. mellifera* showed that the transcriptional expression levels of GS were particularly high in the 4th and 7th instar larvae, whereas the relative expression level of *GP* was lower in the 6th instar and newly emerged adults (Łopieńska-Biernat et al., 2018). Our experiments showed that the glycogen content was higher at the last stage of the brown planthopper larvae (Figure 1). The *TPS* developmental pattern of brown planthoppers from the first instar to the adult on day 15 was determined. The results showed that the expression level of *TPS* was constant and there was no significant difference between the ages (Chen et al., 2010). By aligning the sequences, we learned that they indicated the expression level of *TPS1* in the brown planthopper. Our results also indicate that trehalose can be detected from the 4th instar to the adult stage of brown planthopper, and its content increases before molting or emergence (Figure 1). Therefore, we speculated that energy reserves are accumulated to be used during metamorphosis as well as to provide reserves for the new adult. In addition to trehalose, the glycogen content increased in the late 5th and adult stages (Figure 1) and may also be similar to the effect of trehalose. The developmental expression pattern of *TPS* can also be detected in other insects. The highest level of *TPS* expression at one age of larval stage was found in *A. mellifera* and *Bactrocera minax* (Xiong et al., 2016; Łopieńska-Biernat et al., 2018). Differently, the highest expression was found in the adult stage of *M. domestica* (Zhang D. W. et al., 2019) and in the early pupa of *H. vitessoides* (Chen et al., 2020). In addition, the glucose content was low in the 4th instar, rich in the early 5th instar, and remained stable and abundant in the adult population. It is thus speculated that the newly emerged adult of brown planthoppers needs to consume more energy to maintain life and reproduction. In harsh conditions such as hunger and cold, glucose, trehalose, and glycogen are transformed into each other thereby maintaining the balance in the insect body and continuing life (Shi et al., 2017; Zhang Y. et al., 2019). Therefore, carbohydrates are an important source of energy and play an important role in insects.

The insect fat body is an important place for trehalose synthesis, like the liver in mammals (Candy and Kilby, 1959; Murphy and Wyatt, 1965; Friedman, 1968; Tang et al., 2018). Therefore, many studies have found that *TPS* mRNA expression can be detected in fat bodies (Cui and Xia, 2009; Xu et al., 2009; Chen et al., 2010; Tang et al., 2010; Xiong et al., 2016). Moreover, the expression level of TPS in the fat body is far higher than that in other tissues such as the epidermis, midgut, and the Malpighian tube in *Tribolium castaneum*, *B. minax*, *H. vitessoides*, and *N. lugens* (Chen et al., 2010, 2018, 2020; Xiong et al., 2016). In this experiment, only the expression levels of three *TPSs* in the head, leg, wing bud and cuticle of brown planthopper were determined. The *TPS* gene was found to be expressed in all the tissues examined (Figure 2), and similarly, the expression level of TPS was also detected in the head of *H. vitessoides* (Chen et al., 2020). Conversely, almost no *TPS* expression was detected in the epidermis of the brown planthopper (Chen et al., 2010). The reason for the contradiction between our results is that the age of the test insect is different, and the *TPS* expression in their tissues may thus, be different. In this study, the expression level in the wing bud was higher than that in the other three tissues (Figure 2), indicating that *TPS* is closely related to the energy consumption in brown planthopper flight. Similar studies are also available in other insects, for example, hemolymph trehalose is an important substrate for carbohydrate storage and flight in locusts (Wegener et al., 2010). Research has found that trehalose catabolism is lower in the flight muscles when the locusts are resting, but rapidly increases during flight (Jutsum and Goldsworthy, 1976; Van der Horst et al., 1978; Wegener, 1996; Mentel et al., 2003). In order to utilize muscle metabolism, trehalose must be hydrolyzed to glucose by TRE, and so, the potential activity of TRE in the flying muscle of locusts is high (Worm, 1981; Vaandrager et al., 1989; Becker et al., 1996; Wegener et al., 2003; Liebl et al., 2010). Therefore, we speculate that *TPS* will be expressed in the locust flying muscle to maintain the synthetic catabolism of trehalose. TRE activity in the homogenate of flying muscles from cockroaches, moths and locusts has been observed (Zebe and Mcshan, 1959; Gussin and Wyatt, 1965; Gilby et al., 1967; Candy, 1974).

In fact, in addition to trehalose synthesis in the fat body, the basic cells of the fat body are fat cells, characterized by the presence of many lipid droplets. The intermediate metabolism of most insects occurs in this organ including lipid and carbohydrate metabolism, protein synthesis, amino acid, and nitrogen metabolism. Therefore, fat bodies play an important role in insect life, and are a dynamic organization involving a variety of metabolic functions (Arrese and Soulages, 2010). Firstly, we checked the inhibitory effect of dsRNA, and the results showed that it can effectively inhibit the expression of target genes (Figure 3), which can be used in subsequent experiments. However, after the injection of dsTPS3, the relative expression levels of *TPS1* and *TPS2* were not different from those of the control group, indicating that there may be a compensation effect between the three *TPS* genes (Figure 3). Then in our experiments, differential genes between the dsTPS3 and dsTPSs groups were screened by DGE analysis. The experimental results showed 2632 identical differential genes between the two groups, of which 1048 were upregulated and 1564 were downregulated. Yang et al. (2017) carried out RNA interference on *TPS1* and *TPS2* in the brown planthopper, and also detected the expression of differential genes in the two treatments. The number of upregulated and downregulated genes in the dsTPS1 group were found to be more than those in the dsTPS2 group, and there were 212 upregulated genes and 268 downregulated genes in both groups. Compared with our results (Figure 4), the upregulated genes in the dsTPS3 group differed from those in the dsTPS1 and dsTPS2 groups by 703 and 933, respectively, and the downregulated genes differed by 1469 and 1808, respectively. In addition, the upregulated genes in the dsTPSs group differed by 610 and 840, respectively, from the dsTPS1 and dsTPS2 groups, and the downregulated genes differed by 1269 and 1608. From this point of view, inhibition of *TPS3* gene expression or mixed inhibition of the three *TPS*, results in greater differential gene expression in the brown planthopper compared to that with inhibition of *TPS1* and *TPS2* alone. This result may indicate that the physiological roles of the three *TPS* genes in brown planthopper are different. In microorganisms, TPS and TPP are required for trehalose biosynthesis in yeast and filamentous fungi, including *Fusarium graminearum* (Song et al., 2014; Liu et al., 2019). Three mutants were obtained, a TPS mutant, a TPP mutant, and a double deletion of TPS and TPP (TPS-TPP mutant). None of these three mutants could synthesize trehalose. Compared with the wild type (WT), the lower number of specific genes in the TPS-TPP mutant indicated that double deletion of TPS and TPP resulted in altered expression of most genes detected in the TPS or TPP mutants. Differential genes were analyzed by the MIPS function, and the functional classification showed that compared with the WT, the differentially expressed genes with annotation function could be divided into 18 categories. The metabolic pathway constitutes the most affected of the three mutants, and the TPP mutant has the most genes in all categories. In addition, there were a large number of unknown genetic products (Song et al., 2014). Similar to our results, most functions of differential genes were aggregated by sugar metabolism, amino acid metabolism, and lipid metabolism, and the function of some genes was unknown. It is worth noting that the relative differences in nucleotide and hormone metabolism are nil among dsTPS1, dsTPS2 and dsTPS3 groups. So *TPS3* may play a greater role in general metabolic pathways, which are physiologically important to insects. In summary, when *TPS* expression is affected, the expression levels of genes involved in metabolism in the organism are upregulated or downregulated.

Whether in *T. castaneum* or the *B. minax*, the trehalose content was reduced after injection of dsTPS (Xiong et al., 2016; Chen et al., 2018). In addition, the same experimental results were achieved in *M. domestica* and *H. vitessoides* (Zhang D. W. et al., 2019; Chen et al., 2020). Although the trehalose content increased significantly after 48 h of the mixture of dsTPS1, dsTPS2, and dsTPS3 in our experiments, the trehalose content was decreased significantly after 72 h (Figure 6). The reason for our analysis was that the brown planthopper was stimulated in the early stage of RNAi, which made it synthesize trehalose to maintain normal metabolism, and its interference effect was more obvious. We also tested the changes in glycogen and glucose levels and found that the levels of both sugars were first increased and then decreased (Figure 6). In the study on *B. minax* and *H. vitessoides*, upon *TPS* inhibition by RNAi, the change in trehalose content and glucose content was the opposite, and the glucose content was increased compared with the control group (Xiong et al., 2016; Chen et al., 2020). Combined our experimental results indicate that upon blocking trehalose synthesis, insects will consume glucose and glycogen to replenish energy. However, not all insects show the same experimental results; for example, glucose fluctuated after treatment in *T. castaneum*, but showed no significant difference (Chen et al., 2018). It is thus suggested that there may be species-specific differences between different insects, which may also be related to experimental methods, suggesting that the ability of glucose to compensate for trehalose may not be so obvious. When *Maruca vitrata* was starved, the expression level of *TPS* was significantly higher than that in the feeding process, whereas the result of *TRE* was opposite to that of *TPS*. Moreover, after injection of dsTPS, the trehalose content decreased with the prolongation of injection time, but increased gradually after injection of dsTRE (Al Baki et al., 2018). In summary, the above results indicate that when the expression level of insect *TPS* was inhibited, trehalose synthesis was inhibited and the glucose content was increased.

In addition to the important role of the *TPS* gene in the trehalose metabolism pathway, there are several key genes in this pathway including *TRE*, Hexokinase (*HK*), Glucose-6-phosphate isomerase (*G6PI*), and so on. In previous studies on brown planthopper *TPS1* and *TPS2*, the expression levels of some genes in the pathway after RNAi showed a decrease (Yang et al., 2017). Similar results were obtained in this study. However, the difference was that although the expression levels of these genes were decreased in the early stage of RNAi, they were increased after 72 h, and some genes were even significantly higher than those in the control group (Figure 7). In particular, the *GS* gene showed a significant increase after RNAi (Figure 7), indicating that glycogen synthesis was promoted in the early stage of injection. Similarly, the expression of six chitin synthesis-related genes such as hexokinase 2 and glutamine-fructose-6-phosphate aminotransferase was suppressed at 48 and 72 h after dsTPS injection (Chen et al., 2018). Additionally, *TPS* silencing inhibited the expression of three key genes in the chitin biosynthesis pathway and exhibited 52% death and abnormal phenotypes in *B. minax* (Xiong et al., 2016). In *H. vitessoides*, silencing of *TPS* suppressed the expression of six key genes in the chitin biosynthesis pathway and one key gene related to lipid catabolism (Chen et al., 2020). In addition to the genes of the trehalose metabolic pathway, several genes were involved in chitin synthesis. From this point of view, inhibition of *TPS* expression by RNAi, would not only affect the energy metabolism of insects, but would also have different effects on chitin metabolism. Of course, some studies have detected the chitin content and deformity rate of insects after dsTPS injection such as in *T. castaneum*, *B. minax*, and *H. vitessoides* (Xiong et al., 2016; Chen et al., 2018, 2020). In our study, the results strongly suggest that TPS is essential for the normal growth and development of brown planthopper, providing a reference for further studies on the key genes involved in chitin and lipid metabolism to control insect development.

In summary, in *N. lugens*, the change in carbohydrate content was related to the molting of nymphs, and three *TPS* genes were expressed in the adult head, feet, wings, and epidermis. After inhibiting the expression of *TPS3* or *TPSs*, glucose accumulation was promoted in the early stage, and subsequently *N. lugens* consumed trehalose for energy.

## Data Availability Statement

The sequencing data has been deposited into the Sequence Read Archive (accession: PRJNA658271, https://www.ncbi.nlm.nih.gov/sra/PRJNA658271).

## Author Contributions

BT, CL, and C-DX contributed to the conceptualization, project administration, and supervision. S-SW, Y-KL, Y-JL, and G-YL contributed to the investigation and validation. S-SW, G-YL, CL, and BT contributed to the writing of the original draft. All authors contributed to the article and approved the submitted version.

## Conflict of Interest

The authors declare that the research was conducted in the absence of any commercial or financial relationships that could be construed as a potential conflict of interest.
