# Supplementary material for: Regulation of Carbohydrate Metabolism by Trehalose-6-Phosphate Synthase 3 in the Brown Planthopper, Nilaparvata lugens
Source: Front Physiol. 2020 Sep 17;11:575485. doi: 10.3389/fphys.2020.575485 (PMC7527630; doi:10.3389/fphys.2020.575485)

**Supporting information**

**S1 Figure 1. Changes in trehalose synthesis metabolic pathways after injection of dsTPS1, dsTPS2, dsTPS3 and dsTPSs.** Red: upregulated; green: downregulated.


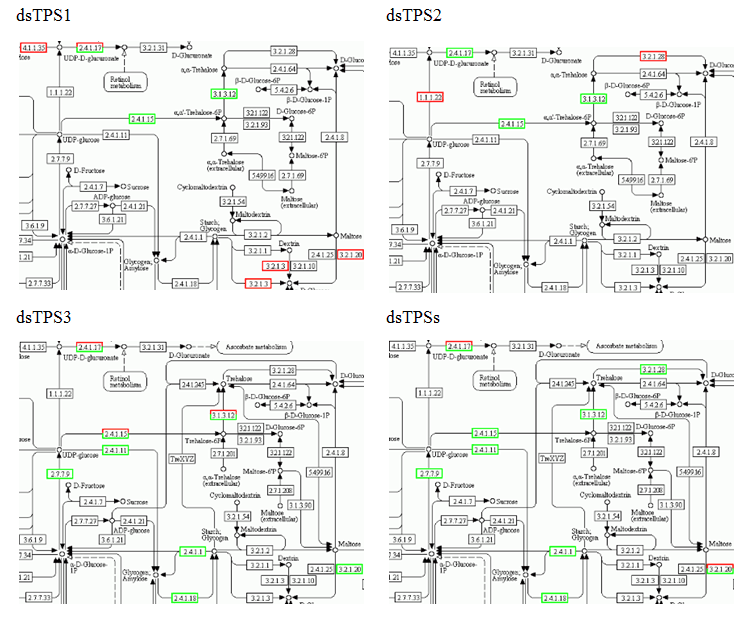

Supplement: Supplementary file 1 [file Data_Sheet_1.DOCX]
